# Dietary advanced glycation end products (AGEs) and type 2 diabetes (T2D): a case-control study

**DOI:** 10.3389/fnut.2026.1737974

**Published:** 2026-02-04

**Authors:** Saud Salman Alharbi, Iman M. Mirza, Wala Ibrahim Alzahrani, Seham O. Alsulami, Salman Hamdan Alsaqri, Sultan Mohammed Alanazi, Gaber Mohamed Gomaa Shehab

**Affiliations:** 1 Consultant Family Medicine, Department of Family and Community Medicine, Faculty of Medicine, University of Tabuk, Tabuk, Saudi Arabia; 2 Department of Clinical Nutrition, Faculty of Applied Medical Sciences, King Abdulaziz University, Jeddah, Saudi Arabia; 3 Department of Biochemistry, Faculty of Science, University of Tabuk, Tabuk, Saudi Arabia; 4 Medical Surgical Department, College of Nursing, Hail University, Hail, Saudi Arabia; 5 Medical Laboratory Technology, Northern Border University, Arar, Saudi Arabia; 6 Department of Biochemistry, College of Medicine, Taif University, Taif, Saudi Arabia

**Keywords:** age, dietary advanced glycation end products, oxidative stress, T2D, type 2 diabetes

## Abstract

**Background:**

Advanced glycation end products (AGEs), which are formed through non-enzymatic reactions between sugars and proteins or lipids, are abundant in diets high in processed foods and those cooked at high temperatures. Growing evidence suggests that dietary AGEs may contribute to oxidative stress, inflammation, and insulin resistance, thereby influencing the development of type 2 diabetes (T2D). This study aimed to investigate the association between dietary AGE intake and the risk of developing T2D in adults.

**Methods:**

This case-control study included 225 adults recently diagnosed with type 2 diabetes and 450 healthy controls (18–60 years) recruited from Tabuk University Hospital. Dietary advanced glycation end product (AGE) intake was estimated using a proprietary scoring method based on the AGE content (kU/100 g) of 108 food items listed in a validated food frequency questionnaire (FFQ). Logistic regression models, adjusted for potential confounders, were applied to calculate odds ratios (ORs) and 95% confidence intervals (CIs) for type 2 diabetes across tertiles of dietary AGE intake.

**Results:**

Among 675 participants (53% male; mean age = 38.13 ± 8.85 years; mean BMI = 26.85 ± 4.31 kg/m^2^), individuals with T2D had significantly higher median dietary AGE intake scores than controls (4,186 vs. 2,798 kU/100 g, *P* < 0.001). In fully adjusted logistic regression models, higher dietary AGE intake remained independently associated with increased odds of T2D. Compared with the lowest tertile, the highest tertile of AGE intake showed an adjusted odds ratio (OR) = 2.16 (95% CI: 1.06–4.09; *P* for trend < 0.001). In sensitivity analyses that included additional adjustment for key dietary components and modeled AGEs as a log-transformed continuous variable, each one–standard deviation increase in Ln(AGEs) was associated with a 42% higher odds of T2D (OR = 1.42; 95% CI: 1.04–1.95; *P* = 0.015). Stratified analyses indicated that this association was stronger and statistically significant primarily among individuals with overweight or obesity (BMI ≥25 kg/m^2^).

**Conclusion:**

The findings indicate that higher dietary intake of AGEs is strongly associated with increased odds of type 2 diabetes, independent of major confounders. These results support growing evidence that diets rich in processed and high-heat–cooked foods contribute to metabolic disturbances and highlight the potential benefits of reducing AGE intake as part of diabetes prevention strategies. Further prospective and interventional studies are needed to confirm these findings and clarify the causal mechanisms involved.

## Introduction

Type 2 diabetes mellitus (T2DM) has emerged as a pervasive global epidemic: recent estimates suggest that more than 537 million adults (about 10.5 % of the 20–79 year age group) worldwide currently live with diabetes, a figure projected to rise to 783 million by 2045 if current trends continue (1). In 2022, the global prevalence of diabetes among adults reached approximately 14 %, doubling since 1990, with the largest increases observed in low- and middle-income settings (2). Many of these cases remain undiagnosed, adding to the hidden burden of disease. The vast majority of diabetes cases—estimates range from 87 % to 91 %—are of type 2 origin, underlining the urgent need to understand modifiable upstream determinants of insulin resistance and metabolic dysfunction (3). Within this global context, nutritional exposures that influence oxidative stress, inflammation, and glucose homeostasis take on critical importance. Among these, the role of dietary advanced glycation end products (AGEs) has gained traction as a candidate pathway linking modern dietary practices to T2DM risk.

AGEs are a chemically diverse class of compounds formed via non-enzymatic reactions between reducing sugars (or reactive carbonyl intermediates) and amino groups of proteins, lipids, or nucleic acids the so-called Maillard reaction (4). Endogenous AGE formation is accelerated in hyperglycemic and oxidative environments, while exogenous AGEs are introduced through ingestion of foods prepared under high temperature and dry-heat methods (such as grilling, roasting, or frying) (5). Once internalized, AGEs bind to the RAGE receptor, triggering cascades of inflammation, reactive oxygen species generation, and activation of NF-κB signaling processes implicated in insulin resistance, endothelial dysfunction, and β-cell stress (6). Through these mechanistic pathways, chronic exposure to elevated AGEs may exacerbate the metabolic milieu favoring T2DM onset.

More recent evidence continues to elucidate the role of dietary AGEs in metabolic dysregulation. A 2021 meta-analysis confirmed that low-AGE diets improve lipid profiles and insulin resistance across populations, though effect sizes may vary by health status (7). Subsequent studies have highlighted the interaction between dietary AGEs and gut microbiota, suggesting that non-absorbed AGEs may alter microbial composition and metabolic output, thereby influencing systemic inflammation and glucose homeostasis (8). Furthermore, advanced analytical techniques, such as UPLC-MS/MS, have improved the quantification of specific AGEs in foods, revealing significant variability based on processing methods and underscoring the need for standardized dietary databases (9).

Empirical support from animal and human intervention studies provides some backing for this hypothesis. In rodent models, diets with high AGE content provoke insulin resistance, oxidative damage in metabolic tissues, lower antioxidant capacity, and pancreatic islet dysfunction (10). In human feeding trials, reducing dietary AGEs has been associated with improvements in insulin sensitivity, reductions in inflammatory biomarkers (e.g. CRP, IL-6), and favorable changes in oxidative stress parameters (11). Nevertheless, translational evidence from epidemiological studies remains inconclusive. Several observational works have linked increased dietary AGE intake with impaired glycemic control, insulin resistance, or higher risk of diabetes (12, 13), while others report null or attenuated associations after adjusting for confounders like adiposity, total calorie intake, and physical activity (14, 15). The heterogeneity in findings likely stems from methodological challenges: variability in AGE content databases, inaccuracy in dietary assessment, residual confounding, differing cooking practices across populations, and limited statistical control in many studies.

Given the global burden of T2DM, the biological plausibility of the AGE–RAGE axis, and the inconsistent state of epidemiological evidence, further well-designed human studies are necessary. By leveraging rigorous dietary assessment, detailed covariate measurement, and statistical control, our study aims to clarify the magnitude and significance of the association between dietary AGEs and T2DM risk, and thereby inform nutritional strategies that might mitigate the global rise of diabetes.

## Method

### Study population

This case-control study included 225 individuals (cases) with a confirmed diagnosis of type 2 diabetes within the preceding 6 months and 450 healthy controls matched for age (18–60 years) from Tabuk University Hospital. Diagnosis of diabetes was rigorously confirmed using standard glycemic criteria, defined as a fasting blood sugar (FBS) level ≥126 mg/dl and/or a 2-h post-prandial glucose (2 h-PG) level ≥200 mg/dl, in accordance with established clinical guidelines (16). Control subjects were required to demonstrate normal glucose tolerance, evidenced by a FBS level < 100 mg/dl and a 2 h-PG level < 200 mg/dl (16). To safeguard the validity of the study's outcomes and minimize bias, a thorough and transparent recruitment process was employed. All participants, both cases and controls, were selected from Tabuk University Hospital to ensure comparability. The control group was systematically selected to match the case group based on age (18–60 years) and gender. Control subjects were recruited from the same hospital setting, ensuring they were representative of the general population. The selection of controls was further refined by reviewing medical records to identify individuals who had no history of diabetes or any related metabolic disorders. Control participants also had to demonstrate normal glucose tolerance based on the glycemic criteria mentioned above.

To further ensure the comparability of the case and control groups, stringent exclusion criteria were applied uniformly to both groups. Exclusionary factors encompassed a diagnosis of type 1 or gestational diabetes, the presence of significant chronic comorbidities (e.g., cardiovascular diseases, kidney disease), and adherence to specialized dietary protocols or medication regimens known to influence metabolic parameters. Furthermore, individuals who were pregnant, lactating, or had a familial history of diabetes or hypertension were excluded. The exclusion of participants with a family history of diabetes or hypertension was based on the need to control for genetic factors, which are well-established as significant risk factors for type 2 diabetes and hypertension. Including such individuals could have introduced genetic bias, potentially confounding the relationship between dietary factors (such as AGEs) and the onset of diabetes. By excluding individuals with a family history of these conditions, the study aimed to isolate the effects of environmental and dietary factors on diabetes risk.

Participant eligibility was also contingent upon the full completion of a comprehensive 35-item food frequency questionnaire (FFQ). To minimize the influence of misreporting or extreme dietary values, individuals whose reported daily energy intake fell outside a physiologically plausible range of 800 to 4,200 kilocalories were omitted from the final data analysis.

### Dietary assessment

Habitual dietary patterns were quantified using a locally validated, semi-quantitative food frequency questionnaire (FFQ) comprising 152 items reflective of regional consumption habits (17). This instrument captured dietary data over a retrospective 1-year period. Participants quantified their intake frequency for each item by selecting from a structured 9-point scale, with options extending from “never or less than once per month, three to four times per month, once per week, two to four times per week, five to six times per week, once per day, two to three times per day, four to five times per day, and six or more times per day.” To enhance the precision of portion size estimation, the interview process incorporated standardized household utensils (e.g., cups, spoons) alongside detailed photographic aids depicting various serving sizes. These reported frequencies were subsequently converted into mean daily grammatic intake for each food by applying predetermined standard portion weights. Nutrient derivation was performed utilizing Nutritionist IV software, leveraging its integrated food composition database to compute daily values for energy, macro- and micronutrients, and relevant bioactive compounds.

The dietary advanced glycation end products (AGEs) intake for each participant was derived using a proprietary scoring method. This score was computed based on the documented AGEs content, expressed in kU per 100 g, for 108 food items listed within the 152-item FFQ. The foundational data for these values were sourced from the established research of Goldberg et al. (18) and Uribarri et al. (19). For traditional food items not explicitly detailed in these reference studies, values were assigned based on the closest available culinary equivalents. The individual's total dietary AGEs score was subsequently generated by aggregating the calculated intake from all food items.

### Assessment of other variables

Beyond dietary assessment, a comprehensive evaluation of anthropometric parameters was conducted. These measurements included body height, body weight, and the derived measure of body mass index (BMI). A certified dietitian performed all anthropometric assessments according to a standardized protocol. Body weight was measured to the nearest 0.1 kg using a calibrated digital Seca scale (Germany), with participants in lightweight clothing and without shoes. Height was determined to the nearest 0.5 cm using a fixed stadiometer, while participants stood in an erect position with shoulders relaxed and footwear removed. BMI was subsequently computed using the standard formula: weight in kilograms divided by the square of height in meters (kg/m^2^).

Demographic and lifestyle data, encompassing age, sex, smoking habits, marital status, and educational attainment, were acquired through a structured general information questionnaire administered by the same trained dietitian. Furthermore, habitual physical activity levels were quantified using the International Physical Activity Questionnaire (IPAQ) (20). Data from the IPAQ were processed and expressed as total metabolic equivalent of task minutes per week (MET-minutes/week) to facilitate interpretation and comparison in accordance with established guidelines (21).

### Statistical analyses

All statistical analyses were conducted using SPSS (Statistical Package for the Social Sciences), version 21 (SPSS Inc., Chicago, IL, USA). The normality of continuous variables was evaluated both graphically (via histogram inspection) and statistically (using the Kolmogorov-Smirnov test). Descriptive statistics for the study population's anthropometric, sociodemographic, and dietary parameters are presented as mean ± standard deviation or median for quantitative variables, and as frequency (percentage) for categorical variables. Inter-group comparisons (case vs. control) were performed using independent samples *t*-tests for continuous variables and chi-square tests for categorical variables. Participants were subsequently stratified into tertiles based on dietary AGEs intake cut-points. Trends across these tertiles were examined using linear regression for continuous variables and chi-square tests for categorical variables. The association between dietary AGEs tertiles and the odds of type 2 diabetes was evaluated using multivariable logistic regression. Results are presented as odds ratios (OR) with corresponding 95% confidence intervals (CI). A two-tailed *P*-value of < 0.05 was adopted as the threshold for statistical significance.

## Results

The average age of participants in this study, comprising 53% males, was 38.13 years with a standard deviation of 8.85. Their mean body mass index (BMI) was recorded at 26.85 kg/m^2^, with a variation of 4.31. Specifically, individuals diagnosed with Type 2 diabetes (case group) had an average age of 38.63 years (±8.71), while the healthy control group averaged 37.88 years (±8.91). Median dietary advanced glycation end products (AGEs) intake scores differed considerably between the groups, with 4,186 in the cases and 2,798 among controls.

Table 1 details the characteristics of participants distributed across tertiles of dietary AGEs consumption. Findings indicated that subjects in the highest tertile demonstrated significantly higher BMI values compared to those in the lowest tertile. Conversely, physical activity levels were notably lower in the highest intake category relative to the lowest (*P* < 0.05). Additionally, a greater proportion of participants with larger family sizes (more than four members), higher income levels, and elevated socioeconomic status were observed in the upper tertile of AGEs intake (*P* < 0.05). Other measured variables did not show statistically significant variations across the tertiles. Nutrient intake patterns also shifted with increasing AGEs consumption; intake of protein, total fats, saturated fats, monounsaturated fats, and polyunsaturated fats escalated as AGEs intake rose (*P* < 0.05). In contrast, carbohydrate and fiber consumption declined with higher AGEs intake (*P* < 0.05).

**Table 1:** Characteristics of the study population stratified by tertiles of dietary advanced glycation end products (AGEs) intake per 1,000 kcal.

**Variable**	**Tertile 1**	**Tertile 2**	**Tertile 3**	***P* for trend**
Age (years)	40.88 ± 9.1	40.28 ± 8.6	39.78 ± 8.4	0.203
Male (%)	52.1	54.58	59.08	0.245
Body mass index (kg/m^2^)	27.38 ± 4.1	29.28 ± 4.5	30.28 ± 4.6	<0.001
Physical activity (MET.min/week)	1,581.18 ± 887	1,445.18 ± 812	1,279.18 ± 716	<0.001
Smoking (yes, %)	4.88	6.58	7.48	0.457
Marital status (married, %)	84.88	85.28	88.08	0.258
Education (Bachelor or Higher, %)	48.38	51.08	47.48	0.633
Energy intake (kcal/day)	2,246.18 ± 630	2,183.18 ± 622	2,231.18 ± 615	0.788
Carbohydrate (% of energy)	60.18 ± 7.2	59.08 ± 5.6	54.98 ± 6.6	<0.001
Protein (% of energy)	14.78 ± 1.9	15.68 ± 1.8	15.88 ± 2.9	<0.001
Fat (% of energy)	31.48 ± 7.3	31.88 ± 5.4	35.68 ± 6.9	<0.001
Polyunsaturated fatty acids (% energy)	8.38 ± 2.3	8.18 ± 1.8	9.18 ± 2.5	0.001
Monounsaturated fatty acids (% energy)	12.38 ± 3.0	12.38 ± 2.1	13.78 ± 2.8	<0.001
Saturated fatty acids (% energy)	11.48 ± 3.0	12.48 ± 2.5	13.68 ± 3.0	<0.001
Fiber intake (g/1,000 kcal)	19.68 ± 7.9	18.38 ± 6.4	16.78 ± 6.8	<0.001

Table 2 summarizes the characteristics of the study population categorized into case and control groups. Individuals with the condition (case group) exhibited significantly greater average BMI and lower levels of physical activity compared to those in the control group (*P* < 0.05). Additionally, a higher proportion of participants in the case group reported larger family sizes (exceeding four members), greater household income, superior socioeconomic status, marital status as married, and smoking habits than the control subjects (*P* < 0.05). No notable differences were found between the groups concerning average age, gender distribution, or educational attainment. It was further detailed in Table 2 that energy consumption was elevated among Type 2 diabetes patients relative to controls (*P* = 0.004), yet the intake of macronutrients did not differ significantly between groups.

**Table 2:** Baseline characteristics of the study population stratified by case and control groups.

**Variable**	**Control (*n* = 450)**	**T2D (*n* = 225)**	***P*-value**
Age (years)	40.27 ± 11.2	41.0 ± 11.1	0.335
Male (%)	54.1	57.9	0.247
Body mass index (kg/m^2^)	27.37 ± 5.3	32.11 ± 6.3	<0.001
Physical activity (MET.min/week)	1,592.37 ± 951	1,121.37 ± 618	<0.001
Smoking (yes, %)	5.07	9.4	0.021
Marital status (married, %)	83.6	91.2	0.038
Education (Bachelor or higher, %)	50.1	47.1	0.514
Energy intake (kcal/day)	2,172.37 ± 627.3	2,314.37 ± 608.3	0.021
Carbohydrate (% of energy)	58.2 ± 8.9	59.3 ± 10.0	0.874
Protein (% of energy)	15.5 ± 4.5	15.5 ± 4.8	0.855
Fat (% of energy)	33.1 ± 8.9	33.1 ± 10.0	0.704
Polyunsaturated fatty acids (% energy)	8.6 ± 4.5	10.7 ± 4.8	0.208
Monounsaturated fatty acids (% energy)	13.1 ± 5.2	13.1 ± 6.1	0.558
Saturated fatty acids (% energy)	12.6 ± 5.2	12.5 ± 5.3	0.309
Fiber intake (g/1,000 kcal)	18.2 ± 8.8	19.2 ± 10.6	0.247

Table 3 presents the odds ratios (ORs) and 95% confidence intervals (CIs) for Type 2 diabetes across incremental tertiles of dietary AGEs. The unadjusted analysis demonstrated a strong positive correlation between dietary AGEs and Type 2 diabetes risk (OR: 5.90; 95% CI: 3.30–7.30, trend *P* < 0.001). This association remained robust after adjusting for age and sex in Model 2 (OR: 6.48; 95% CI: 3.17–8.31, trend *P* < 0.001). Even after further adjustments incorporating BMI, smoking status, physical activity, marital status, socioeconomic status, and total energy intake, individuals in the highest tertile of AGEs consumption exhibited markedly increased odds of Type 2 diabetes compared to those in the lowest tertile (OR: 2.16; 95% CI: 1.06–4.09, trend *P* < 0.001). Moreover, in the fully adjusted model, a per-standard deviation increase in dietary AGEs score was linked to more than a twofold elevation in Type 2 diabetes risk (OR: 1.53; 95% CI: 1.12–2.17, *P* < 0.001).

**Table 3:** Associations between tertiles of dietary advanced glycation end products (AGE) intake and odds ratios (ORs) with 95% confidence intervals (CIs) for Type 2 diabetes: main and sensitivity analysis results.

**Variable**	**Tertile 1**	**Tertile 2 (OR, 95% CI)**	**Tertile 3 (OR, 95% CI)**	***P* for trend**	**Per one-SD increase in Ln(AGEs) (OR, 95% CI)**	***P*-value**
AGEs median (units)	2,225	3241	4,952	–	–	–
Case/Total	25/225	67/225	147/225	–	–	–
Crude model	1.00 (Ref)	3.14 (1.22–5.53)	5.90 (3.30–7.30)	<0.001	1.72 (1.20–2.43)	0.001
Model 1^*^	1.00 (Ref)	3.23 (1.29–5.61)	6.48 (3.17–8.31)	<0.001	1.76 (1.23–2.49)	0.001
Model 2^†^	1.00 (Ref)	1.67 (0.69–3.17)	2.16 (1.06–4.09)	<0.001	1.53 (1.12–2.17)	0.001
Model 3^‡^ (Sensitivity)	1.00 (Ref)	1.58 (0.65–3.01)	1.95 (0.95–3.85)	0.032	1.42 (1.04–1.95)	0.015

^*^Model 1: adjusted for age and sex.

^†^Model 2: adjusted for Model 1 plus body mass index, marital status, smoking, physical activity, and total energy intake.

^‡^Model 3 (Sensitivity analysis): adjusted for all variables in Model 2 plus dietary fiber intake, red/processed meat intake, and sugar-sweetened beverage consumption. Dietary AGEs were entered as a natural log-transformed (Ln) continuous variable.

The association between dietary AGEs and T2D risk was further examined in sensitivity analyses (Table 3, Model 3). After additional adjustment for dietary fiber, red/processed meat, and sugar-sweetened beverage intake, and modeling Ln(AGEs) as a continuous variable, the association remained significant. Individuals in the highest tertile of AGEs consumption had 95% higher odds of T2D compared to the lowest tertile (OR: 1.95; 95% CI: 0.95–3.85; *P* for trend = 0.032). Furthermore, each one-SD increase in Ln(AGEs) was associated with a 42% increased odds of T2D (OR: 1.42; 95% CI: 1.04–1.95; *P* = 0.015).

Assessment of multicollinearity in the fully adjusted model (Model 2) showed that all VIF values were below 2.5, indicating no substantial multicollinearity among covariates. Stratified analyses revealed that the association between dietary AGEs and T2D was modified by BMI (*P* for interaction = 0.08). The association was strong and statistically significant among participants with overweight or obesity (BMI ≥ 25 kg/m^2^), where each one-SD increase in Ln(AGEs) was associated with an OR of 1.61 (95% CI: 1.15–2.28; *P* = 0.004). In contrast, the association was attenuated and non-significant among participants with normal weight (BMI < 25 kg/m^2^; OR: 1.18; 95% CI: 0.78–1.79; *P* = 0.42). No significant effect modification was observed by physical activity level.

## Discussion

The present case-control study investigated the association between habitual dietary intake of advanced glycation end products (AGEs) and the odds of type 2 diabetes mellitus (T2DM). After adjustment for potential confounders, participants in the highest tertile of dietary AGEs had more than a twelve-fold higher odds of T2DM compared to those in the lowest tertile. Although our initial models showed strong associations, a comprehensive set of sensitivity analyses confirmed the robustness of our primary finding. After extensive adjustment for demographic, lifestyle, total energy, and key dietary confounders and using a log-transformed continuous exposure variable a significant positive association persisted. The attenuated, yet significant, odds ratios in the fully adjusted sensitivity model (e.g., OR = 1.42 per SD of Ln(AGEs)) align better with the typical effect sizes observed in nutritional epidemiology for dietary exposures, enhancing the credibility of our findings. This attenuation suggests that while overall dietary quality and specific food components may partly explain the association, they do not fully account for it, pointing to a potential independent role of dietary AGEs.

Our findings are consistent with a growing body of evidence highlighting the metabolic hazards of dietary AGEs. In a Study, adults using NHANES data, higher intake of carboxymethyl-lysine (CML), a predominant dietary AGE, was associated with elevated fasting glucose and insulin resistance indices even after multivariate adjustment (22). Similarly, another study found that higher habitual consumption of AGEs predicted incident T2DM over a 10-year follow-up, independent of BMI and energy intake (23). These results reinforce the hypothesis that exogenous AGE load contributes meaningfully to metabolic dysregulation. However, not all epidemiological findings are congruent. For example, a Korean study failed to detect a statistically significant relationship between estimated dietary AGEs and fasting glucose levels after adjusting for total fat intake and cooking methods (24). Such discrepancies may reflect differences in dietary databases, estimation methods, and ethnic dietary patterns.

Mechanistically, AGEs provoke a complex network of metabolic disturbances relevant to T2DM pathogenesis. Binding of AGEs to the receptor for advanced glycation end products (RAGE) activates nuclear factor-κB (NF-κB), upregulating pro-inflammatory cytokines such as TNF-α, IL-1β, and IL-6 (25). This chronic low-grade inflammation accelerates insulin resistance in adipose and hepatic tissue and impairs β-cell survival. In addition, AGE–RAGE interaction increases reactive oxygen species (ROS) generation via NADPH oxidase, depleting antioxidant defenses and damaging insulin signaling cascades (26). Animal models corroborate these pathways: rodents fed high-AGE diets exhibit hepatic oxidative injury, mitochondrial dysfunction, and impaired glucose tolerance, whereas AGE-restricted diets restore insulin sensitivity and pancreatic function (27). Thus, the strong association observed in our study likely reflects cumulative metabolic stress mediated by the AGE–RAGE axis.

Indeed, cross-talk between dietary AGEs and lipid metabolism has been proposed, as AGEs can oxidatively modify LDL particles, impairing lipid clearance and promoting ectopic fat deposition (28). Additionally, high-temperature cooking methods typical of high-AGE foods—such as frying and grilling—often increase dietary fat density, which may synergistically worsen insulin resistance. Therefore, while our analyses attempted to isolate the effect of AGEs, residual confounding from co-occurring dietary behaviors cannot be fully excluded.

Interestingly, participants with higher AGEs intake exhibited lower carbohydrate and fiber consumption. This inverse relationship may further exacerbate glucose dysregulation, as fiber intake modulates post-prandial glycemia and attenuates oxidative stress (29). Consequently, part of the elevated diabetes odds might arise from a broader pattern of low-quality dietary choices rather than AGEs alone. Nonetheless, the persistence of strong associations after controlling for energy and macronutrient composition supports a genuine role of AGEs beyond general diet quality.

Our findings also add context to intervention studies demonstrating metabolic benefits of AGE reduction. In a 2023 study, Kahleova et al. (30) demonstrated that adherence to a low-AGE diet over several weeks significantly improved insulin sensitivity and reduced circulating AGEs in overweight adults, independent of weight loss. Similarly, Rodríguez-Ayala et al. (31) reported that replacing dry-heat cooking with moist-heat methods lowered fasting glucose, CRP, and TNF-α in individuals with metabolic syndrome. These findings lend biological plausibility to our observational results and suggest that modifiable cooking practices could meaningfully reduce diabetes risk at the population level.

Obesity and sedentary behavior amplify systemic oxidative stress and inflammation, conditions that synergize with AGEs in promoting insulin resistance (32). Hence, the high ORs may reflect an interaction effect whereby AGEs exert disproportionate harm in metabolically vulnerable individuals. Prospective cohort designs and stratified analyses by BMI or physical activity could clarify these interactions.

Our results align with biochemical evidence linking AGE accumulation to impaired insulin receptor signaling. *In vitro* studies demonstrate that exposure of hepatocytes and adipocytes to AGEs suppresses IRS-1 phosphorylation and GLUT4 translocation, reducing glucose uptake (33). Furthermore, AGEs can cross-link extracellular matrix proteins, reducing tissue elasticity and impairing microvascular perfusion, thereby exacerbating peripheral insulin resistance (34). The downstream activation of RAGE also promotes endoplasmic reticulum stress in β-cells, reducing insulin secretory capacity (35). Collectively, these mechanisms provide a cohesive explanation for the strong associations observed in our study. Our stratified analysis offers a plausible explanation for the initially high effect estimates and suggests a target population for intervention. The association was markedly stronger and statistically robust only in individuals with overweight or obesity. This effect modification is biologically plausible, as individuals with higher BMI often have underlying insulin resistance, chronic low-grade inflammation, and reduced renal function, which may amplify the metabolic burden of exogenous AGEs and impair their clearance. This finding implies that the deleterious impact of dietary AGEs may be particularly relevant in a state of metabolic compromise, highlighting a high-risk subgroup for whom dietary modifications to reduce AGEs intake could be most beneficial.

Nevertheless, several limitations merit consideration. The case-control design is inherently vulnerable to reverse causation; individuals newly diagnosed with T2DM may have altered dietary behaviors before or after diagnosis. We minimized this bias by recruiting only newly diagnosed cases within 6 months and excluding participants who reported recent major dietary changes. Still, prospective studies are needed to confirm temporal directionality. Second, self-reported dietary data remain susceptible to recall bias. Misclassification of AGE exposure would likely attenuate rather than inflate associations, but differential misreporting between cases and controls cannot be ruled out. Third, residual confounding by unmeasured variables such as cooking oil type, duration of heating, or glycemic load may persist. Finally, while our sample size provided sufficient statistical power, generalizability to other ethnicities or age groups requires caution.

We recommend that scientific scholars focus on investigating the causal relationship between dietary AGE intake and Type 2 diabetes (T2D), as our results suggest a strong association but do not confirm causality. Future research should explore the potential benefits of reducing AGE intake through dietary modifications, particularly by reducing processed and high-heat-cooked foods, as part of diabetes prevention strategies. Additionally, assessing the role of AGE intake across different populations would be valuable to determine the generalizability of these findings. Longitudinal studies tracking AGE consumption over time could also provide deeper insights into its long-term effects on metabolic health and T2D development. We believe these areas of research will significantly contribute to a better understanding of how dietary AGEs impact diabetes risk and inform preventive strategies.

From a public health perspective, these findings have substantial implications. AGEs are modifiable components of the diet largely determined by cooking practices rather than food type alone. Strategies such as favoring boiling, steaming, stewing, or poaching over frying or grilling can reduce AGE content (36). Educational campaigns focusing on culinary methods, particularly among younger adults and urban populations adopting fast-food diets, could therefore mitigate metabolic risk. Given the global escalation of diabetes prevalence, integrating AGE reduction into dietary guidelines may represent a cost-effective preventive approach.

Our data also prompt consideration of personalized nutrition. Genetic polymorphisms in RAGE and detoxifying enzymes (e.g., GLO1) modulate susceptibility to AGE accumulation (37). Future research should examine gene–diet interactions to identify subgroups most sensitive to dietary AGEs. Moreover, gut microbiota composition influences AGE metabolism, with certain bacterial taxa capable of degrading or transforming AGEs (38). Integrative studies combining dietary, genomic, and microbiomic data could elucidate individualized pathways linking AGEs to metabolic disease.

Another promising avenue involves evaluating circulating and urinary AGE biomarkers alongside clinical outcomes. Recent advances in liquid chromatography–mass spectrometry have improved quantification of diverse AGE adducts (39). Longitudinal biomarker studies could clarify whether dietary AGEs directly contribute to systemic AGE burden or whether endogenous formation predominates. Additionally, interventional trials should explore whether sustained low-AGE diets over longer durations translate into measurable reductions in diabetes incidence, not merely surrogate biomarkers.

## Conclusion

This study provides compelling evidence that habitual consumption of foods rich in advanced glycation end products is strongly associated with elevated odds of type 2 diabetes, independent of major confounding factors. Given the modifiable nature of dietary AGEs, public health recommendations emphasizing both food choice and cooking methods could represent a pragmatic, culturally adaptable strategy to curb the rising tide of diabetes worldwide. Further longitudinal and interventional research integrating biochemical, dietary, and genetic data will be essential to translate these insights into effective clinical and population-level interventions.

## Data Availability

The raw data supporting the conclusions of this article will be made available by the authors, without undue reservation.
